# Simulation of Mass Transport and Phase Transition through Anodic Alumina Membranes Using a Lattice Boltzmann Method

**DOI:** 10.1021/acs.langmuir.5c05693

**Published:** 2026-04-29

**Authors:** Javad Sodagar-Abardeh, Thomas Loimer

**Affiliations:** Institute of Fluid Mechanics and Heat Transfer, 27259 TU Wien, 1060 Vienna, Austria

## Abstract

We present a two-phase multirelaxation-time lattice Boltzmann framework to investigate mass transport in nanoporous membranes under transitional flow conditions. Simulations for the flow through one pore are compared with experimental data for the permeation of low molecular-weight hydrocarbons through anodic alumina membranes with a pore size of 45 nm. Knudsen numbers range between 0.2 and 1.2 for pressures between 1 and 6 bar at room temperature. The model incorporates a modified Peng–Robinson equation of state, extended cohesive and adhesive interactions, and rarefaction-sensitive relaxation times to simulate confined fluid behavior. Special attention is given to the role of adhesive strength in controlling near-wall dynamics through bounce-back and specular reflection mechanisms. To resolve near-wall density variations, a multilayer adhesive model is introduced, thereby extending the influence of wall adhesion deeper into the channel, smoothing the density gradient, and enhancing surface-dominated transport. The adsorbed layer is partially mobile and contributes to the total mass flux rate. This leads to up to a 12% increase in mass flux for light gases, while having a negligible effect on heavier hydrocarbons due to their intrinsic cohesive dominance. For isobutane, a two-phase simulation captures the nonlinear rise in mass flux due to capillary condensation, which is also observed experimentally. Simulation results for methane, ethane, and propane exhibit strong agreement with experimental permeance data, within 5% for methane and 10–15% for ethane and propane, suggesting correct treatment of rarefied gas dynamics, surface effects, and thermodynamic consistency across a range of hydrocarbon species. These findings show the model’s predictive capability and highlight the critical interplay of viscous flow, Knudsen diffusion, surface adsorption, and phase change in nanoscale gas transport.

## Graphical abstract

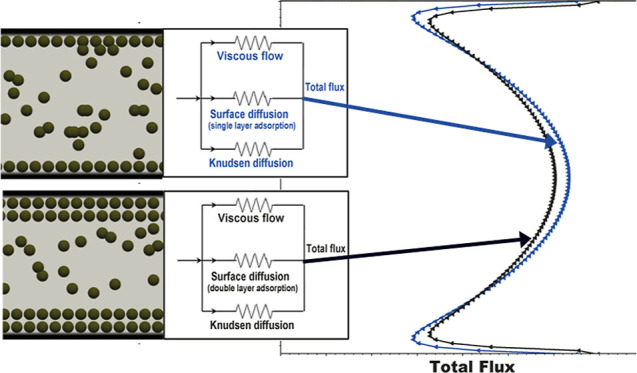

## Introduction

Surface flow via mobile adsorbed layers can significantly contribute to mass transfer in nanoporous materials (2–50 nm) at low pressures or near saturation, where multilayer adsorption occurs.^1–3^ This effect is particularly relevant in applications like gas separation and vapor permeation, where strongly adsorbing species (e.g., hydrocarbons or water vapor) exhibit enhanced transport. Experimental studies demonstrated that in activated carbon, surface flow accounts for a major portion of flux under subcritical conditions, highlighting its critical role in accurate modeling and efficient membrane design.^4,5^ Capillary condensation in nanoporous membranes is another key factor for enhancing separation processes, particularly in gas purification and water and hydrocarbon recovery applications.^6–9^ This phenomenon enables selective condensation of specific vapor components within nanopores, facilitating efficient separation based on differences in condensation behavior. For instance, in natural gas processing, capillary condensation has been utilized to selectively remove heavier hydrocarbons such as propane and butane from methane, improving fuel quality and process efficiency.^6,10^

In the study by Petukhov and Eliseev,^11^ anodic alumina membranes were used to measure gas permeances for various gases (H_2_, He, CH_4_, N_2_, C_2_H_6_, O_2_, Ar, CO_2_, C_3_H_8_, *n*-C_4_H_10_, and *i*-C_4_H_10_) over a Knudsen number range of 0.04–12. Anodic alumina membranes are produced by electrochemically oxidizing metallic aluminum.^12^ The end result is a porous membrane that has parallel, straight, uniformly spaced pores with a very narrow pore size distribution. Hence, these membranes can very well be modeled by a bundle of parallel, straight pores. The experimental data were analyzed using several theoretical models, including the dusty gas, Knudsen, Beskok, and Maxwell first-order slip models, extended to the transition regime while accounting for rarefaction effects on gas viscosity. Experimental investigation of surface flow and capillary condensation^13,14^ in nanoporous media faces major limitations due to the nanometric scale of the phenomena, the difficulty in isolating overlapping transport mechanisms, and the lack of direct methods to measure adsorbed layer mobility or meniscus behavior inside pores. Variations in pore geometry and surface chemistry further complicate interpretation, making it challenging to extract accurate, quantitative data on phase transitions and adsorption behavior.

Monte Carlo methods, particularly Grand Canonical Monte Carlo, offer a powerful way to overcome experimental limitations in studying capillary condensation and surface adsorption by simulating molecular behavior in confined geometries with high resolution.^15^ For example, a multibin Grand Canonical Monte Carlo approach enabled accurate modeling of nonuniform adsorption on pore surfaces,^16^ while simulations of argon in mesoporous silica revealed how pore morphology controls condensation and hysteresis.^17^ Monte Carlo captures equilibrium states but not dynamics;^18^ Molecular Dynamics (MD) was adopted to simulate time-dependent processes such as flow and diffusion at the nanoscale. Molecular dynamics simulations have been instrumental in advancing our understanding of capillary condensation and surface transport in nanoporous materials.^19,20^

Deroche et al.^21^ investigated fluid behavior in subnanoporous structures and revealed a transition from irreversible capillary condensation, typical of larger pores, to a continuous and reversible adsorption regime in smaller pores. This work introduced the concept of “reminiscent capillarity”, capturing the intermediate nature of phase behavior at pore sizes approaching molecular dimensions. Another MD study by Zhang et al.^22^ examined the capillary dynamics of confined water in nanopores, emphasizing the role of precursor films and solid–fluid interactions in modulating the onset and rate of capillary flow. Their findings highlighted the importance of surface forces in shaping quasi-continuum transport behavior at the nanoscale, helping to bridge the scale gap between individual molecular motion and observable flow behavior in experiments.

A key challenge in modeling transport in nanoporous media is bridging the gap between microscopic molecular interactions and macroscopic flow behavior. MD simulation provides detailed insight at the atomic scale but is limited by high computational cost and short time and length scales. These limitations motivate the development of mesoscopic modeling techniques capable of resolving molecular-scale transport without the computational burden of atomistic methods. The Lattice Boltzmann Method (LBM) offers a practical alternative by operating at the mesoscopic level, efficiently capturing key physical phenomena, such as multiphase flow, capillarity, and phase change, over larger domains and longer time scales.^23–27^ As a result, LBM is particularly suitable for simulating gas transport in complex porous domains across mesoscopic length and time scales. Sbragaglia and Succi^28^ challenge the traditional view that the LBM approach is strictly valid only in the continuum regime, corresponding to the limit where the Knudsen number (Kn) becomes increasingly small (Kn → 0). They present a nonperturbative analysis of the Bhatnagar-Gross-Krook kinetic equation for finite Knudsen numbers, demonstrating that LBM can provide semiquantitative results even in the noncontinuum regime, up to Kn∼O(1) . This finding extends the applicability of LBM to transitional regimes where the Knudsen number is not negligible.

Several studies using LBM have successfully addressed the coupled effects of surface flow and capillary condensation in nanoporous systems. Wang and Aryana^29^ developed a coupled framework integrating a modified Peng–Robinson equation of state (EOS) into a multirelaxation-time (MRT) LBM to investigate confined phase behavior and methane transport in slit nanopores, capturing different mechanisms of mass flux under nanoscale confinement. Similarly, Zhao et al.^30^ applied a LBM to simulate water flow through rough nanopores, revealing the critical influence of surface roughness and wettability on nanoscale transport, particularly in regimes where slip and adsorbed layer effects emerge. In a related context, Qin et al.^31^ introduced a contact angle hysteresis model within a two-phase LBM to study drying in porous media, demonstrating how hysteresis and capillary forces govern phase distribution and liquid transport. Collectively, these studies highlight LBM’s capability to bridge mesoscopic modeling with nanoscale interfacial phenomena, providing valuable insights into transport mechanisms that are challenging to resolve experimentally. Furthermore, the variation in local density near solid surfaces plays a critical role in adsorption phenomena within nanopores, particularly for light hydrocarbons, which exhibit more pronounced layering due to weaker intermolecular forces and higher mobility.^32^ Accurate modeling of these effects requires careful consideration of fluid–solid interactions, especially when applying mesoscopic methods such as LBM.

A comprehensive understanding of mass transport at the nanoscale requires integrating both macroscopic and microscopic perspectives. On the one hand, macroscopic parameters, such as the Knudsen number, provide a framework for identifying flow regimes and determining the applicability of continuum or rarefied gas models. On the other hand, microscopic interactions, including cohesive (particle–particle) and adhesive (particle–wall) interactions, govern how molecules behave within confined geometries. By considering both viewpoints, researchers can construct more realistic models that reflect the complex dynamics in nanoporous media.^33,34^ Regarding pressure-driven flow through porous media, three primary transport mechanisms are identified: viscous flow, which dominates when intermolecular collisions govern transport in relatively large pores (low Kn); Knudsen flow, where molecule–wall collisions prevail due to the comparable size of the mean free path and the pore diameter (high Kn); and surface flow, which emerges as a distinct mechanism when adsorbed molecules migrate along pore walls, particularly under near-saturation or strong adsorption conditions.^35,36^ Accurately capturing these overlapping mechanisms is essential for modeling gas and vapor transport in nanoporous materials, especially in applications involving separation, catalysis, or energy storage.

In this work, we develop a two-phase multirelaxation-time (MRT) LBM framework incorporating a modified Peng–Robinson EOS, extended adhesive interactions, and an MRT collision operator to model hydrocarbon transport in nanoporous media. A central objective of this work is, by comparison with experimental data, to resolve and distinguish among three fundamental mass transfer mechanisms: viscous flow, Knudsen diffusion, and surface-driven transport. To achieve this, we systematically calibrate a set of interdependent parameters within the LBM framework, including the fluid–solid interaction strength, the tangential momentum accommodation coefficient, the boundary reflection weight, and the MRT relaxation times governing shear and higher-order nonhydrodynamic modes. A key ingredient in our study is the introduction of a two-layer adhesive interaction scheme, which, to the best of our knowledge, is analyzed here for the first time in the context of its impact on surface flow and near-wall density distribution in rarefied and transitional regimes. This extended interaction model proves particularly important for lighter hydrocarbons, where single-layer formulations underpredict adsorption effects and associated mass flux. Furthermore, the model allows for the simulation of two-phase flows with capillary condensation, as observed for heavier species. In these cases, the formation of a curved interface is captured by employing a specific initialization strategy in combination with an extrapolation-based pressure boundary condition.

In the following, the section “Model description” presents the implementation of the lattice Boltzmann method (LBM) with the multirelaxation-time (MRT) scheme, the formulation and implementation of fluid–fluid and fluid–solid interaction forces and the treatment of rarefied flow effects and near-wall interactions. The section “Validation” validates the equation of state implementation against available property databases and molecular dynamics simulations. The conversion between lattice units and physical units is outlined in section “Unit Conversion”. Subsequently, section “Result and Discussion” presents the computational domain, boundary conditions, and initialization procedures. This section systematically investigates the impact of the two-layer adhesive interaction scheme and parameter calibrations on hydrocarbon transport mechanisms. Key findings on surface-driven transport, capillary condensation, and mass flux distinctions among viscous flow, Knudsen diffusion, and adsorption-driven phenomena are presented and discussed. Finally, the conclusions are summarized.

### Model

#### Governing Equations

The discrete form of the Boltzmann equation,^37^ known as the lattice Boltzmann equation, governs the evolution of the particle distribution function in discrete velocity space and is written as

1 $$f_{i}\left(x+ce_{i}\delta t,t+\delta t\right)=f_{i}\left(x,t\right)+\Omega_{i}\left(f\left(x,t\right)\right)+F_{i}\left(x,t\right)\delta t$$

Here, *f*_*i*_(**x**, *t*) denotes the distribution function associated with the velocity in the *i*th discrete direction **e**_*i*_ at position **x** and time *t*, *c* is the lattice speed defined by *c* = δ*x*/δ*t* where δ*x* and δ*t* are the lattice spacing and time step, respectively. The term Ω_*i*_ is the collision operator, which models the relaxation of the distribution function toward its equilibrium state. The term *F*_*i*_(**x**, *t*) is the discrete external forcing term, accounting for body forces such as interparticle interactions or fluid–solid adhesive forces. Note that the governing equations are presented in dimensional form, all quantities can be expressed in any system of units. Equation 1 describes the dynamics of particles moving and interacting within a discrete lattice, providing a mesoscopic framework for simulating fluid flows.

A two-dimensional nine-velocity model (D2Q9) is employed, with the nine directions given by

$$e_{0}=0,e_{1,3}=\pm e_{x},e_{2,4}=\pm e_{y},e_{5,7}=\pm e_{x}\pm e_{y},e_{6,8}=\pm e_{x}\mp e_{y}$$

where **e**_*x*_ and **e**_*y*_ are the basic unit vectors in a Cartesian coordinate system.

To simulate collisions, the multirelaxation-time (MRT) scheme^38,39^ is applied, where the collision operator is defined as

$$\Omega_{i}\left(f\right)=-M^{-1}S\left(Mf-Mf^{eq}\right)$$

Here, *M* is the transformation matrix that projects the distribution function $f={\left(f_{0},f_{1},...,f_{8}\right)}^{T}$ and the equilibrium distribution function *f* ^eq^ onto the moment space. The relaxation of each moment to its equilibrium state is controlled by the diagonal relaxation matrix *S*, which is given by

$$S=diag\left(\tau_{\rho },\tau_{e},\tau_{\epsilon },\tau_{j},\tau_{q},\tau_{j},\tau_{q},\tau_{s},\tau_{s}\right)$$

Each τ_*i*_ represents the dimensionless relaxation time over which the correponding moment (*Mf*)_*i*_ relaxes to its equilibrium state. Conservation of density and momentum requires that the corresponding dimensionless relaxation times τ_ρ_ and τ_*j*_ are set to unity.^40^ For moments associated with internal energy, the relaxation times are slightly different,^41^ τ_*e*_ = 1.1, τ_ϵ_ = 1.2, hence the associated moments are not conserved. The choices τ_*e*_ = 1.1 and τ_ϵ_ = 1.2 follow a recommendation by Guo et al.^42^ for D2Q9 to damp higher-order kinetic modes and improve numerical stability^39^ under rarefied/confined conditions, while the effective viscosity is controlled independently via τ_*s*_. Although isothermal conditions are assumed, internal energy modes here correspond to higher-order kinetic moments required for numerical stability in the MRT collision operator. The relaxation times τ_*s*_ and τ_*q*_ in the MRT-LBM framework are functions of the Knudsen number and are critical for accurately modeling rarefied gas flows. While τ_*s*_ governs shear stress modes and is related to the effective viscosity, τ_*q*_ is the relaxation time corresponding to the components of energy flux in **e**_*x*_ and **e**_*y*_ direction.^39^ The values of τ_*s*_ and τ_*q*_ and their physical implications are discussed below, see eqs 10 and 11.

The equilibrium distribution function is

2 $$f_{i}^{eq}=w_{i}\rho \left[1+\frac{ce_{i}\cdot u}{c_{s}^{2}}+\frac{{\left(ce_{i}\cdot u\right)}^{2}}{2c_{s}^{4}}-\frac{u\cdot u}{2c_{s}^{2}}\right]$$

where *w*_*i*_ are the weighting factors, (*w*_0_ = 4/9, *w*_1–4_ = 1/9, *w*_5–8_ = 1/36 for the D2Q9 lattice), $c_{s}=c/\sqrt{3}$ , ρ represents the density of the fluid and **u** is the velocity of the fluid. The weighting factors *w*_*i*_ are determined from the requirement that $\sum_{i}f_{i}^{eq}=\rho$ and $\sum_{i}f_{i}^{eq}e_{i}=\rho u$ .^40^ The speed *c*_*s*_ is equal to the isothermal speed of sound for the equation of state of an ideal gas.^40^

#### Interaction Forces

In this study, we adopt a hybrid interaction scheme within the pseudopotential LBM framework^43^ to model dominant transport mechanisms in nanoconfined gas systems. The cohesive (fluid–fluid) force is modeled using a one-layer interaction (nearest and next-nearest neighbors) to reflect the short-range nature of molecular collisions. In contrast, the adhesive (fluid–solid) force includes a two-layer interaction scheme (up to 24 directions) to capture near-wall phenomena such as adsorption, capillary condensation, and surface flow.

In the pseudopotential LBM, pseudoparticles at each lattice node interact according to nonideal potentials, allowing the emergence of phase transition and surface effects. The fluid–fluid interaction (cohesive force) is expressed as

$$F^{cohesive}\left(x,t\right)=-G_{cohesive}\psi \left(x,t\right)\sum_{i\in N_{1}}w_{i}\psi \left(x+e_{i}\delta t,t\right)e_{i}$$

Here, $N_{1}$ refers to the first layer of neighbors in the D2Q9 lattice, *w*_*i*_ are the same weighting factors as reported in eq 2, ψ is the interaction potential, and *G*_cohesive_ controls the strength of fluid–fluid interactions. It is set to *G*_cohesive_ = −1.

The fluid–solid interaction (adhesive force) extends over two interaction layers

$$F^{adhesive}\left(x,t\right)=-G_{1}\psi \left(x,t\right)\sum_{i\in N_{1}}w_{i}S\left(x+e_{i}\delta t,t\right)e_{i}-G_{2}\psi \left(x,t\right)\sum_{j\in N_{2}}w_{j}S\left(x+e_{j}\delta t,t\right)e_{j}$$

Here, $N_{2}$ represents second-nearest or extended neighbors, up to 24 directions, and *S* is a Boolean indicator function equal to 1 at solid nodes and 0 at fluid nodes. The second-layer adhesive term generalizes the interaction stencil, similar in spirit to multirange pseudopotentials proposed for capturing long-range wettability effects.^44^

The parameters *G*_1_ and *G*_2_ control the magnitude and spatial reach of the adhesive force. Specifically, *G*_1_ governs near-wall momentum exchange, while *G*_2_ extends the adhesive interaction further from the wall to capture adsorption effects and enhance the modeling of surface transport mechanisms. This two-layer approach follows the generalization of Sbragaglia et al.,^45^ adapted here to incorporate confinement-sensitive thermodynamic behavior. The weights *w*_*i*_ and *w*_*j*_ are chosen to maintain isotropy of the interaction force. To determine *G*_1_ and *G*_2_, we adjusted their values such that the LBM-predicted near-wall to bulk density ratio closely matched the corresponding ratio observed in the MD data.^15^ This calibration process aims to reproduce the wall–fluid interaction strength in the LBM model to better capture molecular-scale adsorption and layering.

#### Equation of State

In confined environments, both the critical temperature *T*_*c*_ and critical pressure *P*_*c*_ are reduced compared to bulk conditions due to the increased surface area-to-volume ratio and the influence of solid–fluid interactions within the confined space.^46^ This reduction leads to significant deviations in phase behavior, such as suppressed bubble-point pressures and altered phase envelopes.

To account for these confinement effects, we adopt the modified Peng–Robinson equation of state (mPR-EOS) proposed by Wang and Aryana^29^ to model nonideal fluid behavior under nanoconfinement. The mPR-EOS introduces a confinement scaling factor β and a correction term *c* to independently adjust the critical temperature and pressure

3 $$P=\frac{\rho RT}{1-b^{\prime }\rho }-\frac{a^{\prime }\alpha \left(T\right)\rho^{2}-c^{\prime }}{1+2b^{\prime }\rho -b^{\prime 2}\rho^{2}}$$

with *a*′ = *a*/β, *b*′ = *b*/β, *c*′ = *c*/β, and *R* is the specific gas constant. The Peng–Robinson constants are

4 $$a=0.45724R^{2}T_{c}^{2}/P_{c},,b=0.07780RT_{c}/P_{c}$$

where *T*_*c*_ and *P*_*c*_ are bulk critical properties. The parameters *c* and β are defined by

5 $$c=a\left(1-T_{crel}\right),,\beta =P_{crel}/T_{crel}$$

where *T*_crel_ and *P*_crel_ denote the confined critical temperature and pressure, made dimensionless with their bulk counterparts. The temperature-dependent term α­(*T*) accounts for the attractive force correction in the mPR-EOS and adjusts the strength of intermolecular interactions as a function of temperature. It is defined as

$$\begin{array}{l} \alpha \left(T\right)={\left[1+\kappa \left(1-\sqrt{T/T_{c}}\right)\right]}^{2},\\ \kappa =0.37464+1.54226\omega -0.26992\omega^{2}, \end{array}$$

where ω is the acentric factor.

In the LBM framework, the pressure and pseudopotential are related by

$$P\left(x,t\right)=c_{s}^{2}\rho \left(x,t\right)+\frac{1}{2}G_{cohesive}c_{0}\psi^{2}\left(x,t\right)$$

hence, substituting from eq 3 yields for the initialization of the potential

$$\psi \left(x,t\right)={\left[\frac{2}{G_{cohesive}c_{0}}\times \left(\frac{\rho RT}{1-b^{\prime }\rho }-\frac{a^{\prime }\alpha \left(T\right)\rho^{2}-c^{\prime }}{1+2b^{\prime }\rho -b^{\prime 2}\rho^{2}}-c_{s}^{2}\rho \right)\right]}^{1/2}$$

where *c*_0_ is a lattice-specific constant. For D2Q9, *c*_0_ = 6.

#### Knudsen Number

The Knudsen number is a central descriptor of the flow regime, quantifying the degree of rarefaction. In this study, we adopt a local form of the Knudsen number, which varies spatially based on local thermodynamic conditions. The Knudsen number is estimated from

$$Kn\left(x\right)=\frac{k_{B}T}{\sqrt{2}\pi d^{2}H}\left[\frac{1}{P\left(\rho \left(x\right),T\right)}\right]$$

where *k*_B_ is the Boltzmann constant, *T* is the temperature, *d* is the molecular diameter, and *H* is the slit width. The local pressure *P*(ρ­(**x**), *T*) is obtained from the modified Peng–Robinson equation of state, eq 3.

Closely related to the Knudsen-number is the tangential momentum accommodation coefficient (TMAC), denoted here by σ. The TMAC relates the mean of the tangential momentum of incident molecules, ${\mathrm{\tau ̅}}_{i}$ , to the mean of the tangential momentum of reflected molecules, ${\mathrm{\tau ̅}}_{r}$

$$\sigma =\frac{{\mathrm{\tau ̅}}_{i}-{\mathrm{\tau ̅}}_{r}}{{\mathrm{\tau ̅}}_{i}}$$

For purely diffuse reflection, i.e., there is no wall slip, σ = 1, while for purely specular reflection σ = 0. Based on a comprehensive compilation of available experimental reported data, Agrawal and Prabhu^47^ proposed the correlation

6 $$\sigma =1-\log_{10}\left(1+{Kn}^{0.7}\right)$$

for polyatomic gases. This is the correlation used here.

In the lattice-Boltzmann formulation, after the collision step, the outgoing distribution functions from the wall back into the flow domain are given by^48^

$$f_{i}^{t+1}=rf_{i}^{bounce-back}+\left(1-r\right)f_{i}^{specular}$$

where *f*_*i*_^*t*+1^ is the postcollision distribution, *f*_*i*_^bounce‑back^ is the distribution resulting from the bounce-back (diffuse reflection) rule, *f*_*i*_^specular^ is that from specular reflection, and *r* is the mixing parameter. The relation between *r* and the TMAC σ is not straightforward. Rather, Li et al.^41^ found by careful comparisons of implementations of a lattice Boltzmann method with experimental data, solutions of the linearized Boltzmann equations and results from direct simulation Monte Carlo computations for the flow through microchannels, that the mixing parameter is best calculated from

7 $$r=\frac{1}{1+C_{1}\sigma_{v}\sqrt{\pi /6}}$$

where the parameters *C*_1_ and σ_v_ are given by

8 $$\sigma_{v}=\frac{2-\sigma }{\sigma },,C_{1}=1-0.1817\sigma$$

With increasing σ, both σ_v_ and *C*_1_ decrease, hence *r* increases.

Using the above formulation, the parameter *C*_1_ above aligns with the parameter *C*_1_ in the first-order term of the macroscopic second-order slip boundary condition

9 $$u_{s}=C_{1}Kn\left(x\right)\frac{\partial u}{\partial n}-C_{2}Kn{\left(x\right)}^{2}\frac{\partial^{2}u}{\partial n^{2}}$$

The parameter *C*_2_ in the second-order term is also related to the effective viscosity, see further below. For the transitional regime investigated in this study, it is set to *C*_2_ = 0.82, again following Li et al.^41^

As discussed earlier, in the MRT-LBM framework, distinct relaxation times govern different modes of momentum and energy transport. The relaxation time for shear stress modes, τ_*s*_, directly controls momentum diffusion and determines the fluid’s effective viscosity. To incorporate rarefaction effects in the slip and transition regimes, τ_*s*_ is modeled using a Bosanquet-type effective viscosity relation, μ_eff_ = μ_bulk_/(1 + *a* Kn)

10 $$\tau_{s}=0.5+\sqrt{\frac{6}{\pi }}\frac{\left(H/\delta x\right)Kn\left(x\right)}{1+aKn\left(x\right)}$$

where *a* ≈ 2 is a molecular flow correction parameter. For instance, for the flow of a gas through a capillary, writing the mass flow rate as a sum of viscous and molecular flow and estimating the bulk viscosity from kinetic theory of gases yields *a* = 8.1, see eq 3 in ref 49. For real gases and unknown pore space, *a* is an empirical parameter in the Bosanquet-type interpolation; we set *a* ≈ 2 and keep it constant for all gases, while gas- and pressure-dependence enters through Kn­(**x**). This formulation ensures that τ_*s*_ dynamically adapts to the local flow regime state.

To consistently enforce higher-order slip behavior and improve numerical stability, the relaxation time for higher-order nonhydrodynamic (ghost) moments, τ_*q*_, is linked to τ_*s*_ through the expression^38^

11 $$\tau_{q}=0.5+\frac{\pi C_{2}{\left(2\left(\tau_{s}-0.5\right)\right)}^{2}+3}{16\left(\tau_{s}-0.5\right)}$$

where *C*_2_ is an empirical coefficient which corresponds to *C*_2_ in the macroscopic boundary condition, eq 9. This hierarchical formulation allows the MRT-LBM to accurately capture nonequilibrium effects associated with rarefied gas dynamics near solid boundaries.

Other central parameters are related to adhesive strength, *G*_1_ and *G*_2_, which represent the strength and extension of fluid–solid interaction at the channel wall. These parameters govern how fluid accumulates near the surface and influence the density layering effect. Both *G*_1_ and *G*_2_ are not fixed constants; rather, they vary with pressure, temperature, gas type, and the nature of the surface, which defines the degree of momentum exchange. For example, heavier hydrocarbons like butane exhibit stronger adsorption and less layering compared to methane, and therefore require a different arrangement of *G*_1_ and *G*_2_ to replicate molecular behavior seen in MD or experimental data. Also, when temperature or pressure changes, *G*_1_ and *G*_2_ generally need to be modified to maintain similar wall effects. *G*_1_ and *G*_2_ are determined by calibrating them to reproduce near-wall adsorption/layering (near-wall-to-bulk density ratio) reported by molecular simulations for hydrocarbons under confinement. Specifically, *G*_1_ (first-layer adhesion) and *G*_2_ (second-layer extension) are adjusted in a quiescent slit configuration until the LBM-predicted near-wall density enhancement matches the reference MD trend.^50^ Once calibrated for a given gas at room temperature, the values of *G*_1_ and *G*_2_ are kept fixed for the transport simulations of that gas in the considered pressure range.

Since the adhesive interaction strength is related to σ, any change in σ should be accompanied by an appropriate update to *G*_1_ and *G*_2_ and consequently to *r*.

All the key parameters—*G*_1_, *G*_2_, σ, *r*, τ_*s*_, and τ_*q*_—are functions of or influenced by the local Knudsen number. These parameters collectively govern flow behavior in nanoconfined systems, impacting density distribution, wall interactions, and flow regimes.

### Validation

#### Coexistence Curves

The study of phase behavior in confined fluids, particularly in nanoslits, is essential for understanding how confinement alters thermodynamic properties at the nanoscale. Notably, in narrow pores, the critical density (ρ_*c*_) critical temperature (*T*_*c*_), and critical pressure (*P*_*c*_) of a fluid are suppressed compared to their bulk values due to enhanced surface-to-volume ratio and strong solid–fluid interactions.^29^ This section investigates the coexistence phase behavior of methane confined in nanoslits using a coupled version of a two-phase LBM framework with a modified Peng–Robinson EOS which allows for the adjustment of critical properties shift through two parameters, β and *c*.

The mPR-EOS is used in the LBM framework to calculate the pseudopotential at each lattice site, enabling liquid–vapor coexistence under confinement. This coupling allows us to model phase equilibrium in nanoporous systems, with periodic boundary conditions applied to represent bulk-like conditions.

One of the key features of our approach is the ability to vary the adhesive strength between the fluid and solid walls within the two-phase LBM simulation. This parameter plays a crucial role in the fluid’s behavior inside the nanoslit, as the fluid–wall interaction affects the density distribution and phase transition dynamics. For the 5 nm slit pore case, the confinement parameters β and *c* are set according to the relative reduced critical temperature and pressure values (*T*_crel_ ≈ 0.95, *P*_crel_ ≈ 0.83) reported by Wang and Aryana,^29^ see their Figure 1, resulting in β ≈ 0.87 and *c* ≈ 0.05*a*. The adhesive strengths *G*_1_ (primary layer) and *G*_2_ (secondary layer) are then calibrated within the LBM framework to match the methane coexistence densities obtained from eq 3, as illustrated in Figure 1a. By adjusting the adhesive strength, we can model a range of scenarios from weak to strong fluid–wall interactions. This variation directly influences the coexistence curves. This approach is inspired by the findings of Huang et al.,^51^ which also considered the effect of fluid–wall interactions in confined systems by modifying the PR EOS based on LBM simulations.

**Figure 1:**
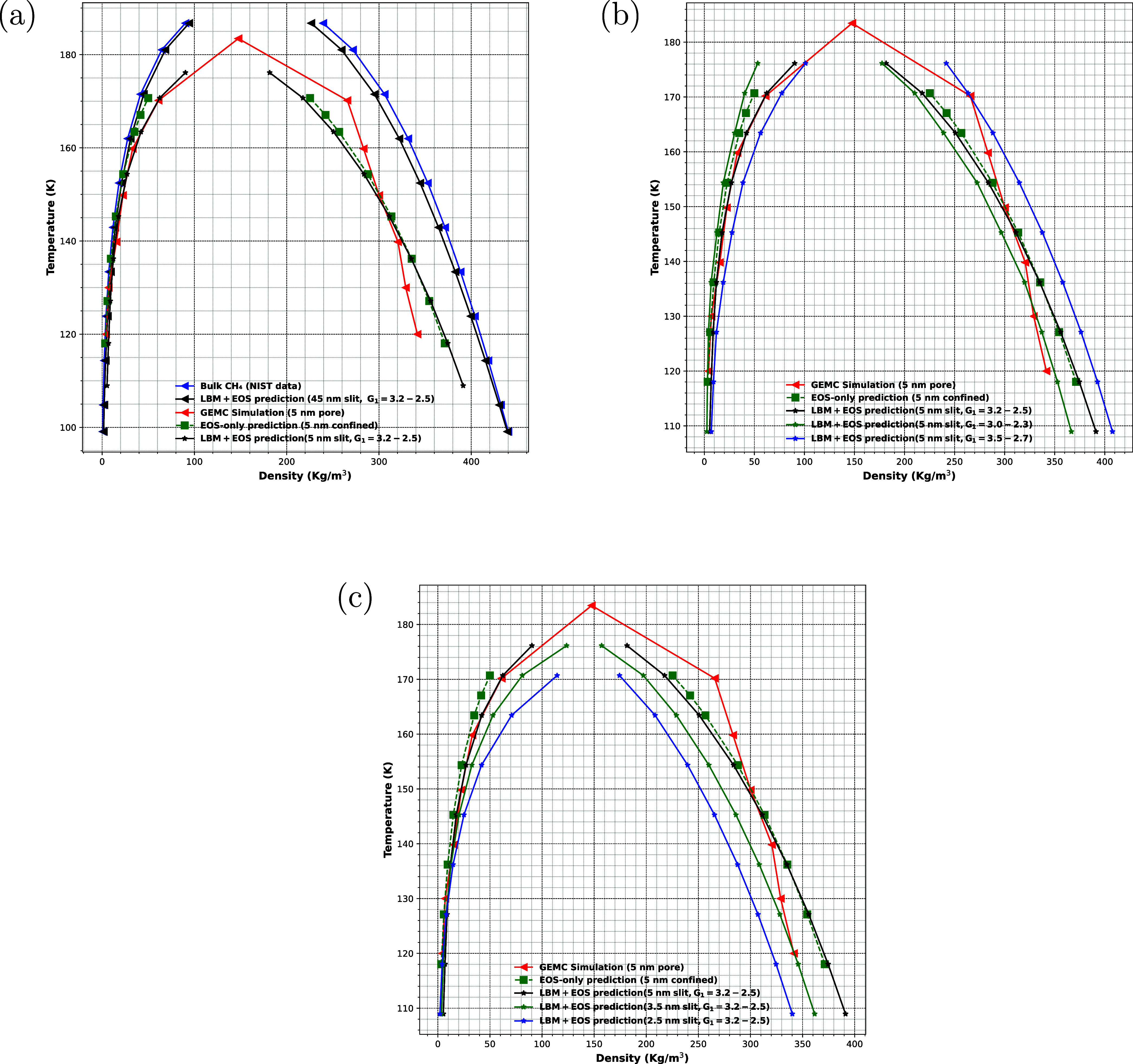
(a) Comparison of coexistence curves from bulk, GEMC, EOS, and LBM; (b) Effect of adhesive strength. (c) Effect of slit width on coexistence curves. For *T*_r_ varying between 0.6 and 0.95 with 0.05 increment, and in all cases *G*_2_ varying between $0.8G_{1}\overset{7steps}{\to }0.4G_{1}$ .

The width of the slit is a key factor influencing phase behavior. Wang and Aryana^29^ demonstrated that methane’s phase behavior varies with slit width by tuning the EOS parameters β and *c*, which are functions of the pore size and directly modify the equation of state to reflect confinement effects. However, their study focused exclusively on validating the EOS results against molecular dynamics (MD) simulation data, without extending this comparison to LBM-based phase diagrams obtained using the coupled mPR-EOS. In the context of LBM modeling, both the width of the nanoslit and the adhesive strengths (*G*_1_ and *G*_2_) play a significant role in shaping the density profile and the fluid–solid interface properties, including the apparent contact angle. It is therefore essential to systematically compare the density phase diagrams and interfacial characteristics generated by the LBM framework to MD reference data, as variations in *G*_1_ and *G*_2_ can substantially alter the equilibrium interface morphology and the predicted coexistence curves. Such direct benchmarking is critical for ensuring that the LBM approach, when coupled with a confinement-corrected EOS, accurately captures the fundamental phase behavior and interfacial physics observed at the molecular scale. Narrower slits, in particular, amplify confinement effects, resulting in pronounced shifts in the coexistence curves and interfacial properties relative to bulk fluid behavior.

We simulate the methane coexistence curve at various reduced temperatures, considering initial conditions where the system is in a mixed phase of liquid and gas. This setup allows us to observe the phase transitions as the temperature increases. Importantly, in order to maintain the same liquid–gas curvature at different temperatures, we find that the adhesive strength must be decreased as the temperature increases. This adjustment ensures that the phase transition dynamics remain consistent across different temperature conditions. The system evolves dynamically, and we track the changes in the coexistence curves over time.

To isolate the effect of fluid–wall interaction strength, subsequent simulations fix β and *c* while varying *G*_1_ and *G*_2_, demonstrating the sensitivity of phase behavior to adhesive forces (Figure 1b). The results show strong sensitivity of the vapor density to changes in both the primary (*G*_1_) and secondary (*G*_2_) adhesive layers, demonstrating the importance of extended fluid–wall interactions in controlling phase separation. Conversely, to study the impact of slit width on phase behavior, *G*_1_ and *G*_2_ are held constant, and β and *c* are varied according to confinement-induced shifts corresponding to different nanoslit widths (Figure 1c).

While the validation is performed at 5 nm to match available molecular simulation data, our target case is a nanoslit of 45 nm. According to Yang et al.,^52^ phase behavior in pores up to 50 nm still deviates from bulk predictions and requires EOS correction. Because direct molecular dynamics (MD) or experimental data at larger pore sizes (e.g., 45 nm) are unavailable, we employ a linear interpolation scheme between the values at 5 nm and bulk fluid conditions at 50 nm, where β = 1 and *c* = 0. Specifically, for any pore width *d* in the range 5 ≤ *d* ≤ 50 nm, the parameters are interpolated. For the 45 nm slit relevant to our study, the linear interpolation yields β ≈ 0.98 and *c* ≈ 0.0056*a*. Hence, from eq 5 we have *T*_crel_ = 0.9944 and *P*_crel_ = 0.9745 for all substances in our study, indicating that confinement effects are greatly diminished but still non-negligible at this scale.

#### Transitional Flow

To validate the accuracy of the present lattice Boltzmann transport model in the slip and transition flow regimes, simulations were performed at Knudsen numbers Kn = 0.1 and Kn = 1.0, and the resulting normalized velocity profiles were compared with DSMC and LBM data reported by Rustamov et al.^53^ The computational domain consisted of 45 × 45 lattice nodes with periodic boundary conditions imposed at the inlet and outlet. A constant external force corresponding to a pressure gradient of 10^–4^ in lattice units was applied to drive the flow. The slip boundary treatment was implemented using a combined bounce-back and specular-reflection scheme, in which the mixing parameter *r* was determined as a function of the Knudsen number according to eqs 6, 7 and 8. For Kn = 1, σ = 0.71 and *r* = 0.48, while for Kn = 0.1 σ = 0.93 and *r* = 0.61.

Figure 2 presents the normalized velocity distributions across the channel for Kn = 0.1 and Kn = 1.0. For Kn = 0.1, the velocity profile remains close to a parabolic shape with moderate slip at the walls, whereas for Kn = 1.0 pronounced slip effects and a flattened velocity profile are observed, indicating the increasing influence of rarefaction. In both cases, the present lattice Boltzmann results show agreement with the DSMC data.

**Figure 2:**
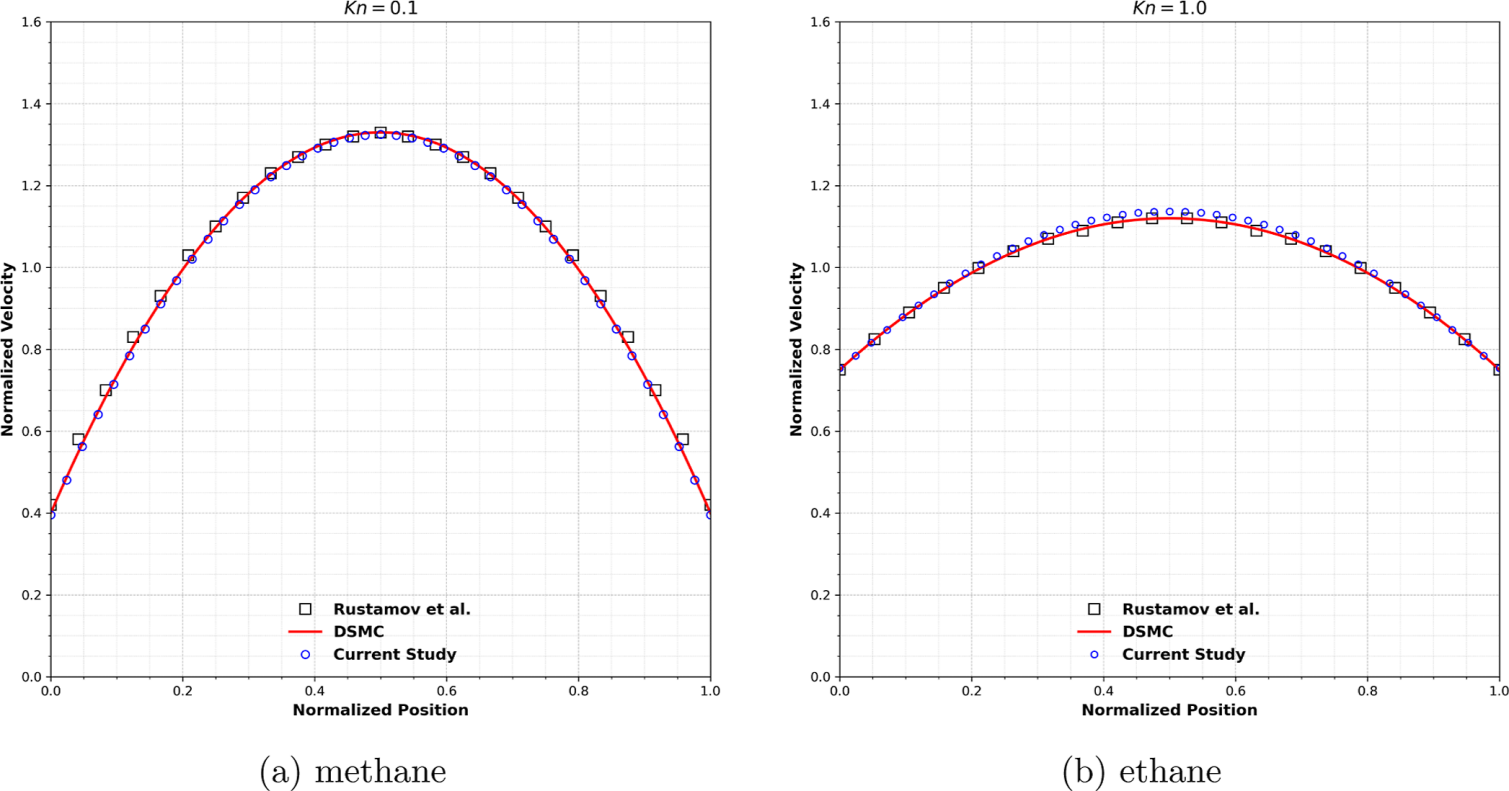
Normalized stream-wise velocity profile for (a) Kn = 0.1 and (b) Kn = 1.

### Unit Conversion

To ensure numerical stability in solving the governing equations, the values of all quantities are expressed in lattice units. In other words, the introduction of such a system of units can be interpreted as a nondimensionalization with respect to the base units of this system. Conversion between lattice units and other unit systems follows standard practices of unit conversion. For the simulations presented here, we set the parameters appearing in the equation of state, eqs 3 and 4, to be

12 $$a=\frac{2}{49}ll^{5}/\left(lmlt^{2}\right),,b=\frac{2}{21}ll^{3}/lm,,R=1ll^{2}/\left(lt^{2}lT\right)$$

where ll, lm, lt and lT stand for the lattice units of length, mass, time and Temperature, respectively. Hence, we have three equations, but four unknowns. In a single-phase flow scenario, lm is chosen arbitrarily, say lm = 0.1 nm. In a two-phase flow scenario with a liquid–vapor interface, the surface tension provides a fourth equation. The value of the surface tension in lattice units is determined by carrying out LBM simulations of liquid drops with different diameters and evaluating the surface tension from Young–Laplace’s equation.

The values for *a* and *b* above are not used directly. From eqs 4 and 12 follows

$$P_{c}=0.059570lm/\left(lllt^{2}\right),,T_{c}=0,072922lT$$

For example, for methane (*T*_*c*_ = 190.564 K, *P*_*c*_ = 4.5992 MPa, R = 518.28 J/(kg K)) and 1 ll = 0.1 nm we have 1 lT = 2613.16 K, lt = 8.5928 × 10^–14^ s, lm = 5.7006 × 10^–29^ kg. (The critical density is 162.66 kg/m^3^ = 2.8533 lm/ll^3^. One *c* = 1163.8 m/s) For isobutane, with γ = 1.1764 × 10^–2^ N/m and a measured value of γ = 0.3 lm/lt^2^ the conversion for the dimension of length is given by γ/*P*_*c*_, hence ll = 0.6437 nm, lT = 5592.4 K, lt = 7.197 × 10^–13^ s and lm = 2.031 × 10^–26^ kg. (ρ_c_ = 2.9613 lm/ll^3^ and *c* = 894 m/s)

## Results and Discussion

### Setup of Simulations

Figure 3 presents the computational domain, boundary conditions, and initialization strategies used for the two distinct flow regimes modeled in this study. The medium used to collect the experimental data, Anodic Aluminum Oxide membranes (AAO-45 nm), consist of parallel pores with a uniform shape. To model this medium, a 2D slit with a width equal to one pore diameter from the experimental setup is considered. The slit is represented by solid wall boundaries. The computational domain extends by 25 lattices at the inlet and the outlet in longitudinal direction, where periodic boundary conditions are applied in lateral direction. These small spaces at the inlet are advantageous for smooth conditions at the inlet and the outlet to the slit. The schematic in Figure 3a shows inlet and outlet conditions for a flow where condensation within the pore occurs.

**Figure 3:**
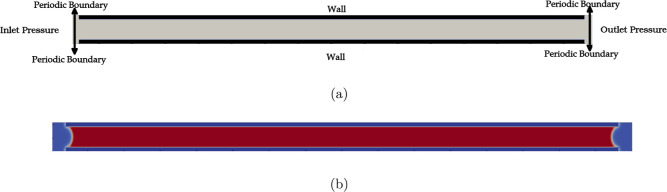
(a) Schematic of domain and boundary conditions for the numerical simulations. (b) The equilibrium state for calculating the capillary pressure.

Different boundary conditions are applied depending on whether condensation occurs or the fluid remains gaseous, see the descriptions on single-phase gas flow and two-phase flow below. For gaseous flows, the pressure gradient is replaced by a body force, periodic boundary conditions are applied in longitudinal direction and only a part of the channel needs to be simulated. For flows with capillary condensation, which only occurs for isobutane, the entire slit must be simulated and pressure boundary conditions are applied. Table 1 reports the mesh sizes and boundary conditions.

**Table 1:** Boundary Conditions at the Inlet and the Outlet in Streamwise Direction and Mesh Sizes of the Simulations

fluid	boundary conditions	mesh size	flow scenario
methane	periodic	1000 × 450	single-phase
ethane	periodic	1000 × 360	single-phase
propane	periodic	1000 × 270	single-phase
isobutane	periodic	1000 × 180	single-phase
isobutane	pressure	150,000 × 70	two-phase

In the simulations, the distinction whether a two-phase flow with condensation or a gaseous, single-phase flow occurs is done by first determining, also in a simulation, the pressure at which condensation within the slit occurs. This capillary pressure is determined by filling the slit with liquid and let it reach equilibrium with the gaseous phase at the inlet and outlet regions.^54^ Depending on whether the inlet pressure is larger or smaller than the such determined capillary pressure, the fluid condenses or gaseous fluid flows through the slit. In the experimental permeance versus mean pressure data (Tables 2–5), the permeance of lighter hydrocarbons such as methane, ethane, and propane exhibit an approximately linear dependence on mean pressure, indicating that their transport remains in the gas phase without phase transition under the experimental conditions. When the inlet pressure exceeds the capillary pressure, capillary condensation occurs, and the pore contains both liquid and vapor phases. Isobutane shows a noticeable kink in the permeance vs mean pressure, signaling the occurrence of capillary condensation within the nanopores. This distinction is critical in determining the appropriate modeling approach.

**Table 2:** Experimental Data for Permeance of Methane^a^

no.	*P*_in_ (bar)	*P*_out_ (bar)	*P*_mean_ (bar)	Knudsen	Permeance (m^3^/(m^2^bar h))	mass flux (kg/(m^2^s))
1	1.305	0.985	1.1450	1.23825	43.9290	0.0153391
2	1.667	0.985	1.3260	1.06923	43.2849	0.0322120
3	1.920	0.985	1.4525	0.97611	43.7158	0.0446013
4	2.210	0.985	1.5975	0.88751	44.4890	0.0594683
5	2.530	0.985	1.7575	0.80671	44.9576	0.0757930
6	2.856	0.985	1.9205	0.73824	43.8273	0.0894778
7	3.370	0.850	2.1100	0.67194	43.9290	0.120795
8	3.798	0.985	2.3915	0.59285	46.2981	0.142112
9	4.270	0.985	2.6275	0.53960	47.0492	0.168649
10	4.610	0.985	2.7975	0.50681	47.4380	0.187643

a Obtained from the authors of ref 11.

**3:** Experimental Data for Permeance of Ethane^a^

no.	*P*_in_ (bar)	*P*_out_ (bar)	*P*_mean_ (bar)	Knudsen	Permeance (m^3^/(m^2^bar h))	mass flux (kg/(m^2^s))
1	1.370	0.980	1.1750	0.81814	31.5388	0.0277436
2	1.540	0.980	1.2600	0.76294	31.6289	0.0399508
3	2.060	0.980	1.5200	0.63244	32.4756	0.0791104
4	2.580	0.980	1.7800	0.54006	33.2103	0.119852
5	3.210	0.980	2.0950	0.45886	34.2357	0.172201
6	3.550	0.980	2.2650	0.42442	35.0198	0.203002
7	4.150	0.980	2.5650	0.37478	36.2821	0.259420
8	4.220	1.520	2.8700	0.33495	37.8465	0.230485
9	4.220	2.040	3.1300	0.30713	38.6898	0.190242
10	4.220	2.700	3.4600	0.27784	38.8425	0.133169

a Obtained from the authors of ref 11.

**4:** Experimental Data for Permeance of Propane^a^

no.	*P*_in_ (bar)	*P*_out_ (bar)	*P*_mean_ (bar)	Knudsen	Permeance (m^3^/(m^2^bar h))	mass flux (kg/(m^2^s))
1	1.510	0.985	1.2475	0.50191	26.5649	0.0433118
2	1.810	0.985	1.3975	0.44804	27.1763	0.0696278
3	2.110	0.985	1.5475	0.40461	27.6407	0.0965694
4	2.412	0.985	1.6985	0.36864	28.7319	0.127329
5	2.710	0.985	1.8475	0.33891	28.8425	0.154511
6	3.115	0.985	2.0500	0.30543	30.5720	0.202228
7	4.144	0.985	2.5645	0.24415	33.5073	0.315045
8	3.850	0.985	2.4175	0.25900	32.1156	0.298121

a Obtained from the authors of ref 11.

**Table 5:** Experimental Data for Permeance of Isobutane^a^

no.	*P*_in_ (bar)	*P*_out_ (bar)	*P*_mean_ (bar)	Knudsen	Permeance (m^3^/(m^2^bar h))	mass flux (kg/(m^2^s))
1	1.333	0.990	1.1615	0.43181	22.8491	0.03272
2	1.710	0.990	1.3500	0.37152	23.3547	0.07021
3	1.710	0.985	1.3475	0.37221	23.7960	0.07203
4	1.930	0.980	1.4550	0.34471	23.6005	0.09361
5	1.930	0.980	1.4550	0.34471	23.9031	0.09481
6	2.291	0.984	1.6375	0.30629	25.3304	0.13822
7	2.296	0.985	1.6405	0.30573	25.2531	0.13822
8	2.628	0.987	1.8075	0.27748	26.3257	0.18037
9	2.980	0.986	1.9830	0.25293	26.7552	0.22274
10	3.222	0.984	2.1030	0.23849	28.2653	0.26411
11	3.366	0.985	2.1755	0.23055	34.4397	0.34236

a Obtained from the authors of ref 11.

#### Single-Phase Flow

For the single-phase gas flow regime, a periodic boundary condition was imposed in the streamwise (*x*) direction, with flow driven by an external body force defined as *F* = Δ*P*/*L*, where Δ*P* is the experimentally measured pressure drop and *L* is the domain length. The simulation domain was initialized with stagnant fluid with a uniform gas density corresponding to the experimental mean pressure and temperature, using values from the NIST Chemistry WebBook.^55^ The spatial resolution is between 6 to 10 lattice units per nanometer, see Table 1. In the longitudinal direction, the resolution is the same, and only a small part of the slit length used in the experiments was modeled. Gas–solid interactions at the wall were modeled using a combination of bounce-back and specular reflection schemes, allowing for an accurate representation of slip flow and tangential momentum exchange in the transitional flow regime. The two-layer adhesive interaction model was calibrated to reproduce the near-wall to bulk density ratio reported in the literature, ensuring consistency with adsorption behavior typical of nanoscale confinement.

#### Two-Phase Flow

For systems where the experimental mass flow data indicated the presence of capillary condensation, such as in the case of isobutane, which deviated from the linear mass flux trend observed for lighter hydrocarbons, a two-phase simulation strategy was employed. The computational domain was equipped with pressure boundaries imposed at both inlet and outlet, where the experimental values of *P*_in_ and *P*_out_ can be directly imposed.^54^ No external body force was applied. The pressure gradient alone was sufficient to drive the flow. The treatment of wall–fluid interactions remained consistent with that used for single-phase simulations.

The initial density field was constructed to contain both liquid and gas phases. Specifically, the slit was filled with liquid, while the inlet and outlet bulk zones were initialized as gas. At the interface a smoothed density profile was prescribed^56^

$$\rho \left(x,y\right)=\rho_{g}+\frac{\rho_{l}-\rho_{g}}{2}\times abs\left\{\tanh\left[\frac{2\left(x-20\right)}{W}\right]-\tanh\left[\frac{2\left(x-780\right)}{W}\right]\right\}$$

where ρ_*l*_ and ρ_*g*_ are the equilibrium liquid and gas densities from Maxwell construction, and *W* = 5 is the interface width. After initialization, the experimental temperature was applied throughout the domain. The system was allowed to relax under isothermal conditions until equilibrium was reached, as determined by a convergence criterion applied to the velocity field. At this stage, a curved gas–liquid interface formed within the slit due to the hydrophilic nature of the walls, and the gas-side pressure was identified as the capillary pressure. This pressure offset arises from interfacial curvature and is consistent with the Young–Laplace relation.^57^

To apply constant-pressure boundaries, a nonequilibrium extrapolation scheme was implemented. The prescribed pressure was first converted to local density using the modified PR-EOS at the specified temperature. This density was then used to evaluate the pseudopotential function, interparticle forces, and the local macroscopic velocity. At boundary nodes, the unknown distribution functions (e.g., *f*_1_, *f*_5_, *f*_8_ at the inlet) were reconstructed using

$$f_{i}=f_{i}^{eq}+\left(f_{\mathrm{i̅}}^{ext}-f_{\mathrm{i̅}}^{eq}\right)$$

where $f_{\mathrm{i̅}}^{ext}$ is the distribution in the opposite direction extrapolated from the interior nodes, and *f*_*i*_^eq^ is the local equilibrium distribution evaluated using the density from the EOS and the velocity obtained from the force balance. The term $f_{\mathrm{i̅}}^{eq}$ is the equilibrium distribution, computed using the same local density and velocity. This formulation avoids spurious reflections or velocity suppression near the pressure boundaries.

For cases involving capillary condensation, the number of lattices is significantly increased to correspond to the physical scale of the liquid–gas interface. This high resolution is necessary to resolve the meniscus location and the diffuse interface accurately, despite the increased computational cost.

### Comparison of Simulations with Experiments

Figure 4 presents a quantitative comparison between the ratio of mass flux to pressure difference (the mass flux is defined as the measured mass flow rate divided by the cross-sectional area of the pores) predicted by LBM and experimental measurements for methane (CH_4_), ethane (C_2_H_6_) and propane (C_3_H_8_) through anodic alumina membranes with a nominal pore diameter of 45 nm. For all three gases, the ratio of mass flux to pressure difference increases monotonically with mean pressure, consistent with expectations for the transitional flow regime. The agreement between simulation and experiment is excellent for methane. For ethane and propane, while the simulations correctly reproduce the overall trend, they slightly overpredict this ratio. This overprediction may be attributed to the idealized representation of the wall–fluid interaction in the simulation model, which does not account for surface roughness or adsorption heterogeneity present in real materials.

**Figure 4:**
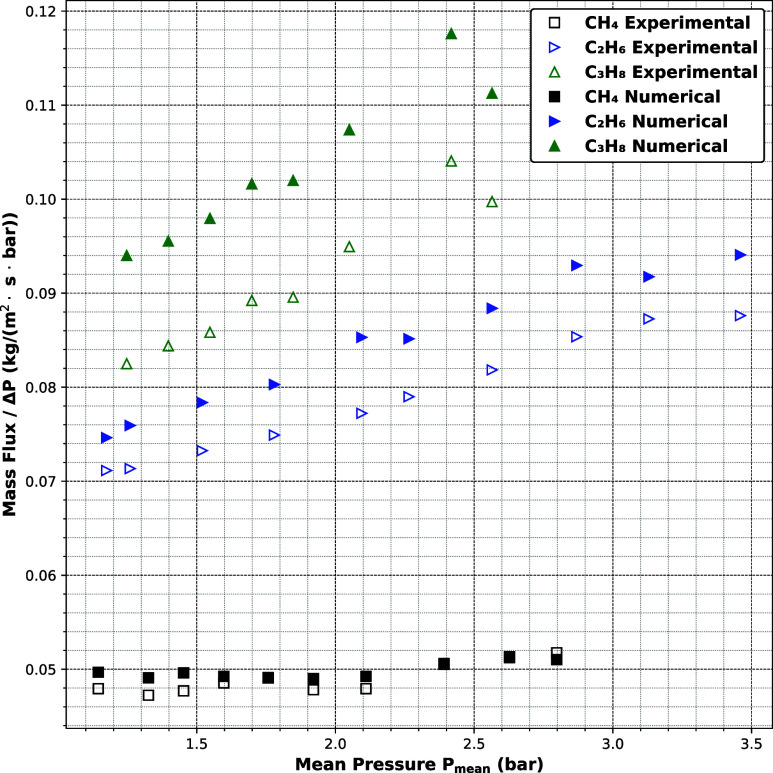
Comparison between the numerical and experimental ratio of mass flux to pressure difference for different hydrocarbons through anodic aluminum oxide (AAO-45 nm).

Figure 5 shows the ratio of mass flux to pressure difference for isobutane (*i*-C_4_H_10_) as a function of inlet pressure. Unlike the lighter hydrocarbons, the flux curve here is distinctly nonlinear, exhibiting a sharp increase for *P*_in_ approaching the saturation pressure. This nonlinearity signals the onset of capillary condensation, which is captured by the simulation through the implementation of two-phase boundary conditions and initialization. After the equilibrium state according to a two-phase flow simulation was reached, different inlet pressure conditions were imposed based on the reported data for isobutane.

**Figure 5:**
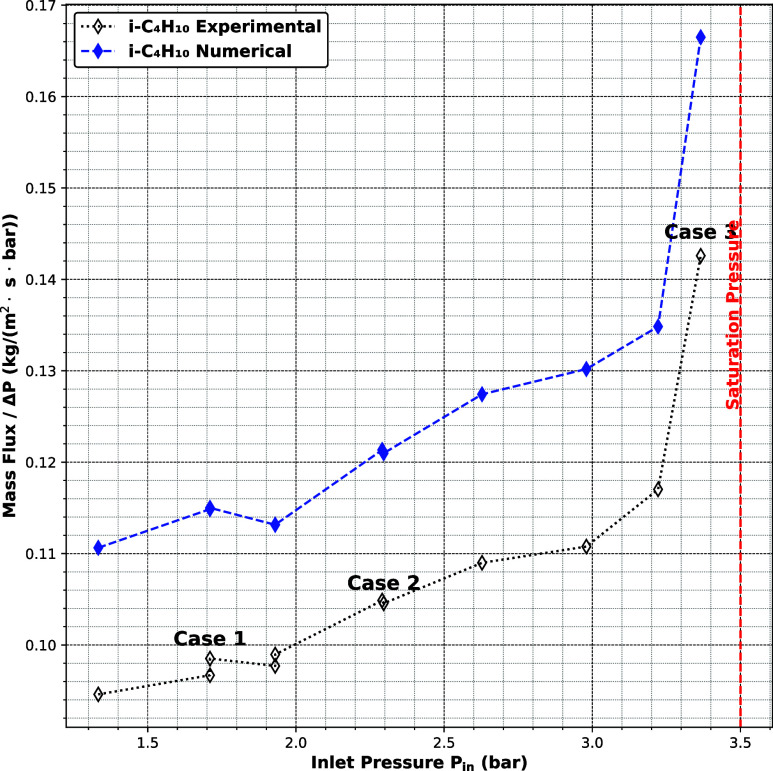
Comparison between the numerical and experimental ratio of mass flux to pressure difference for isobutan through anodic alumina (AAO-45 nm).

For *P*_in_ < *P*_capillary_, after the equilibrium, the pressure gradient destabilizes the gas–liquid interface, resulting in continuous evaporation of the liquid phase. Within the LBM framework, this evaporation is inherently modeled through the evolution of the distribution functions, as mass transfer from the liquid to the gas phase causes a local decrease in density and gradual retreat of the liquid region, without explicit interface tracking. In other words, the liquid plug initially present in the slit evaporates, and eventually, only vapor remains in the pore, and a single-phase flow regime is established.

However, when *P*_in_ > *P*_capillary_ but remains below the saturation pressure, the capillary forces are sufficient to retain liquid within a portion of the slit. The system evolves toward a two-phase steady state, in which a segment of the pore remains filled with liquid, and the strongly curved menisci bounding the segment prevent evaporation of the liquid. In the steady state, mass is transported through the pore; hence, vapor constantly condenses at the upstream meniscus of the liquid segment, and the liquid evaporates at the downstream meniscus. This sustained phase coexistence is the result of a dynamic balance between capillary pressure, interparticle interactions, wall adhesion, and the applied pressure gradient. This behavior is supported by the simulation results shown in Figure 6, where three representative case studies are presented. In Case 1 and Case 2, the initial gas–liquid interface eventually disappears, indicating that the imposed pressure gradient leads to full evaporation. In Case 3, however, a persistent liquid segment remains in the center of the channel, demonstrating a stable two-phase configuration due to capillary retention. The modeling strategy for each regime should be selected based on the relationship between the inlet pressure and the capillary pressure, which is first estimated from an equilibrium simulation involving a curved vapor–liquid interface. If the inlet pressure is lower than the capillary pressure, the system remains in a single-phase gas state. Otherwise, condensation occurs within the slit due to confinement-induced phase separation.

**Figure 6:**
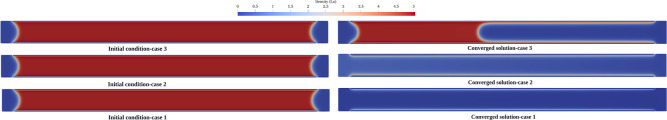
Simulation results of density distribution for the cases in Figure 5 at the initial and steady-state conditions.

Although the extrapolation pressure boundary condition strategy with two-phase initialization demonstrates excellent qualitative agreement with the experimental behavior of *i*-C_4_H_10_, the absolute deviation remains higher compared to lighter hydrocarbons (see Figure 7). This discrepancy may stem from limitations in the model assumptions related to wall surface properties. In the simulations, the solid walls are assumed to be atomically smooth, allowing for well-defined surface layering and interaction behavior. However, in real nanoporous materials, the surface may exhibit roughness, heterogeneous chemical functionality, or microdefects, which can act as adsorption sites for heavy hydrocarbon molecules. Larger molecules like i-C_4_H_10_, due to their higher surface affinity and greater molecular volume, are more likely to become immobilized or trapped at such defect sites. This physical sticking effect would reduce the measured mass flux experimentally, while the simulation—lacking such immobilization—predicts a higher flow rate. Therefore, the elevated deviation for *i*-C_4_H_10_ may reflect a physical limitation in the surface modeling rather than a failure of the LBM framework itself. Incorporating surface roughness or adsorption kinetics in future models could improve agreement for heavier, more interactive species.

**Figure 7:**
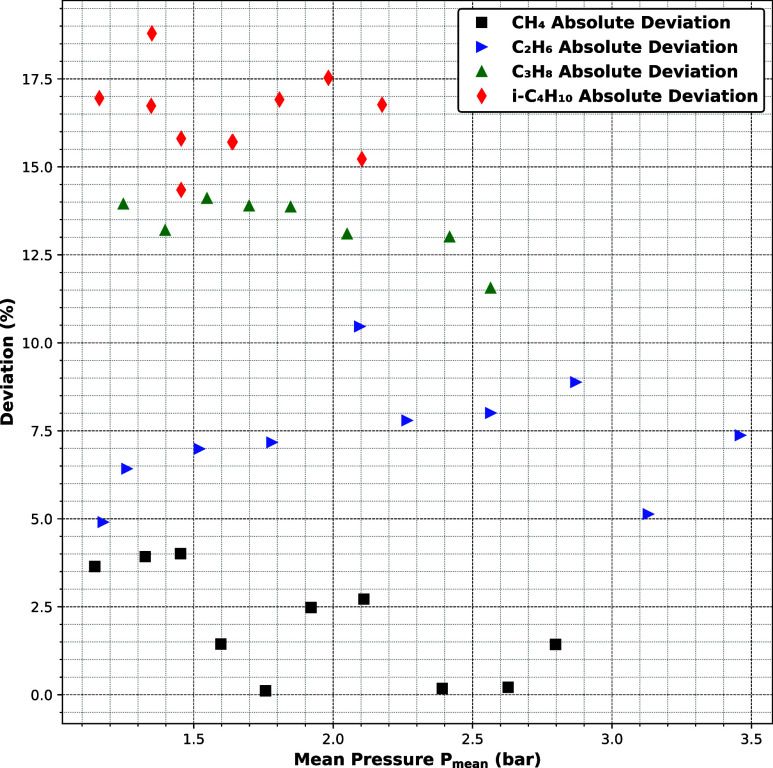
Absolute deviation between experimental and numerical ratio of mass flux to pressure difference for different hydrocarbons through anodic alumina (AAO-45 nm).

Among the hydrocarbons studied using the periodic boundary condition with a body force, CH_4_ shows the lowest absolute deviation from experimental data (Figure 7). This outcome is expected, as methane is the lightest molecule, characterized by high molecular mobility and weak cohesive interactions. Its transport is primarily governed by Knudsen flow in transitional flow regimes, which is inherently less sensitive to near-wall effects. In this case, the assumption of an idealized smooth surface, combined with a density distribution obtained from molecular simulations, seems to be sufficient to accurately capture its flow behavior in nanoporous structures.

As we move to heavier hydrocarbons such as C_2_H_6_ and C_3_H_8_, the deviation from experimental data gradually increases. This can be attributed to two main factors. First, larger molecules interact more strongly with the wall, making the assumption of a perfectly smooth surface less valid. In real porous materials, surface roughness and chemical heterogeneity can lead to enhanced adsorption or localized trapping, reducing the experimentally measured mass flux. These effects are not captured in the current simulations, which assume uniform wall properties and neglect surface irregularities. Second, while the simulations provide access to detailed density distribution and phase profiles, such microscopic data is not available from experiments. As a result, validation is limited to integral quantities like mass flux, and any mismatch in local density gradients or wall-layering effects can contribute to deviation. These limitations become more pronounced with increasing molecular size and interaction with the wall, as seen for C_3_H_8_. Thus, the increasing deviation from CH_4_ to C_3_H_8_ reflects the growing influence of surface effects and modeling assumptions, even in the absence of phase change. It is important to note that the deviation between numerical and experimental results may also be partially attributed to uncertainties in the experimental data. Since the experimental measurements report permeance, possible sources of error include inaccuracies in volumetric flow rate detection, estimation of the effective membrane area, and pressure drop measurement. These uncertainties can be particularly relevant in nanoporous systems, where flow rates are low and surface effects are considerable.

### Double Layer vs Single Layer Interactions

In this section, the effects of single-layer versus double-layer adhesive interactions on flow dynamics within nanochannels are compared. By examining how adhesion influences not only the immediate wall–fluid interface but also extends into adjacent fluid layers, we try to better understand the implications of adhesive interaction range on density distribution and the mechanism of mass transfer.

Figure 8 shows the normalized density distribution of various hydrocarbons—ranging from light (CH_4_, C_2_H_6_) to heavier species (*i*-C_4_H_10_, C_3_H_8_)—within a confined slit geometry up to a quarter of the wall width for the first set of experimental data. The plots illustrate the layering effect near the solid walls due to wall–fluid interactions and how the density distributes from the near-wall region to the bulk region. Differences in molecular structure, as captured by the acentric factor and critical properties in the mPR-EOS, and interaction strength lead to distinct density profiles for each hydrocarbon. The results are obtained using two adhesive interaction models: a single-layer and a two-layer wall–fluid interaction. For light hydrocarbons, the use of a two-layer model leads to more pronounced layering, while the single-layer model underpredicts these effects. In contrast, the heavier hydrocarbons show nearly the same density distribution with both models. This difference is related to the strength of cohesive and adhesive forces: lighter molecules have weaker fluid–fluid interactions and require extended wall attraction to reproduce expected adsorption behavior. Heavier molecules, due to their larger size and stronger intermolecular forces, already form a stable density distribution even with a single interaction layer. These results suggest that modeling light hydrocarbon adsorption in nanoporous systems benefits from a multilayer interaction approach, whereas heavier species can be sufficiently captured with a single-layer adhesive model.

**Figure 8:**
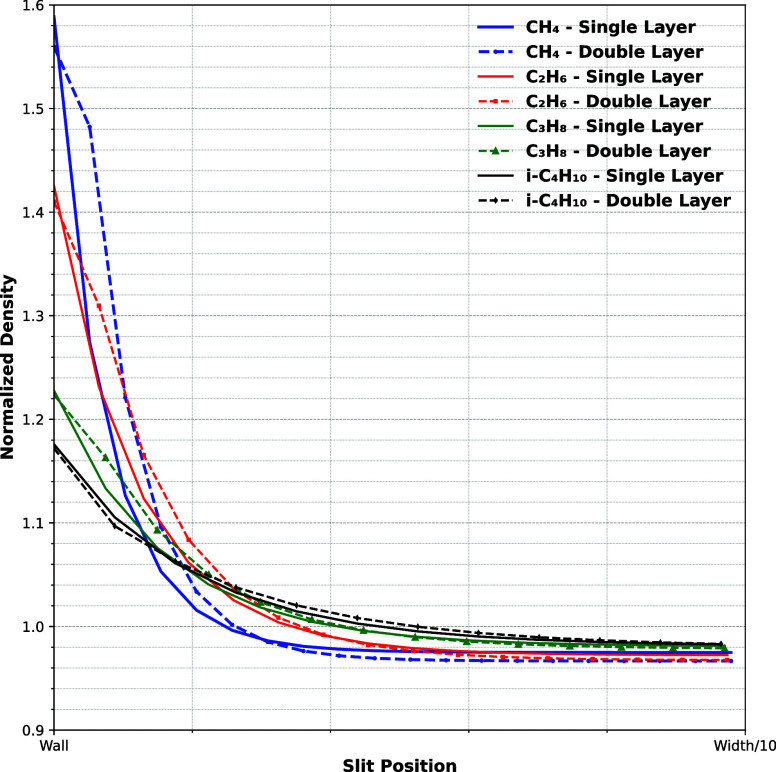
Density distribution of different hydrocarbons confined in a slit pore.

The normalized mass flux (ρ*U*/mass flux) for different hydrocarbons under the first experimental conditions is further analyzed in Figure 9. It can be seen that the total transport consists of three distinct contributions: surface flow, Knudsen flow, and viscous flow—these effects are especially prominent for lighter hydrocarbons such as CH_4_.

**Figure 9:**
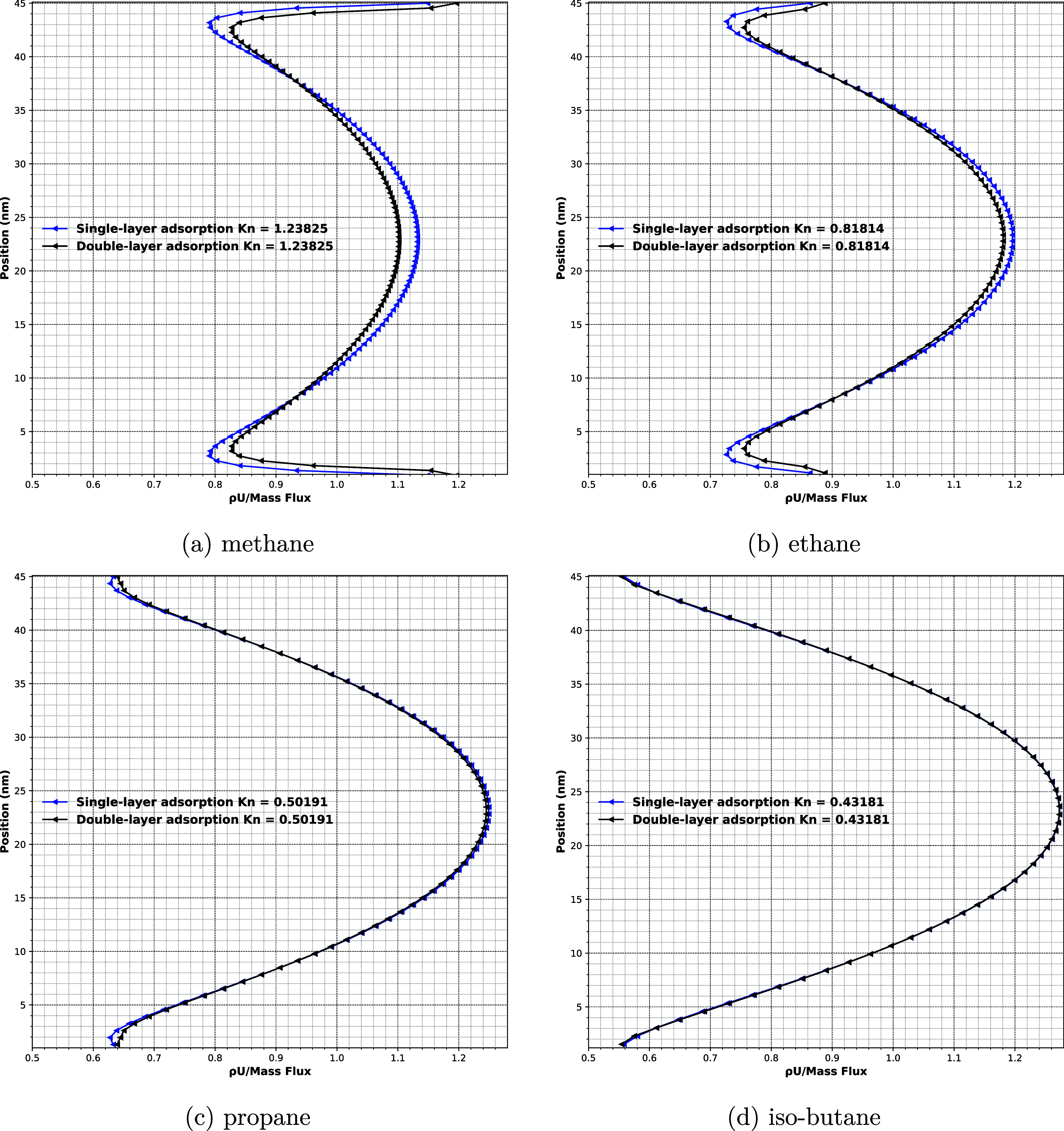
Comparison of mass flux between single and double-layer adsorption scenarios, always for experimental conditions listed as no. 1 in Tables 2–5: (a) methane, *P*_mean_ = 1.145 bar, (b) ethane, *P*_mean_ = 1.175 bar, (c) propane, *P*_mean_ = 1.248 bar, (d) iso-butane, *P*_mean_ = 1.162 bar.

In both the single-layer and two-layer interaction models, the ratio of near-wall to bulk density remains approximately the same (cf. Figure 8). However, for lighter hydrocarbons, the two-layer interaction model modifies the spatial density distribution by extending the layering structure further toward the channel center. While the peak near-wall density remains nearly unchanged, this redistribution shifts a greater fraction of molecules toward the near-wall region, thereby reducing the molecular population in the channel core.

As a result, surface flow increases due to enhanced tangential momentum transfer in the expanded near-wall region. Simultaneously, Knudsen flow becomes more significant because the less population of particles in the slit center increases the local mean free path, promoting molecule–wall collisions over molecule–molecule collisions. Conversely, viscous flow decreases as the continuum-like behavior weakens in the central region. Despite this internal redistribution, the total normalized mass flux is increased under the two-layer interaction model.

This interpretation is further supported by the percentage deviation shown in Figure 10, which presents the increase in mass flux obtained with the two-layer model relative to the single-layer model. The deviation is most pronounced for lighter gases, with CH_4_ showing up to 12% higher flux under two-layer interactions compared to the single-layer case. For C_2_H_6_ and C_3_H_8_, the deviation remains notable, though gradually smaller, reflecting a weakening influence of the interaction range. For the heaviest Hydrocarbon studied, i-C_4_H_10_, the deviation is nearly negligible across all pressure ranges, aligning with the observation that stronger intrinsic cohesive forces dominate the transport behavior. Here, both the density distribution and mass flux profiles converge, indicating that extended adhesive interactions do not alter the dynamics significantly in such cases.

**Figure 10:**
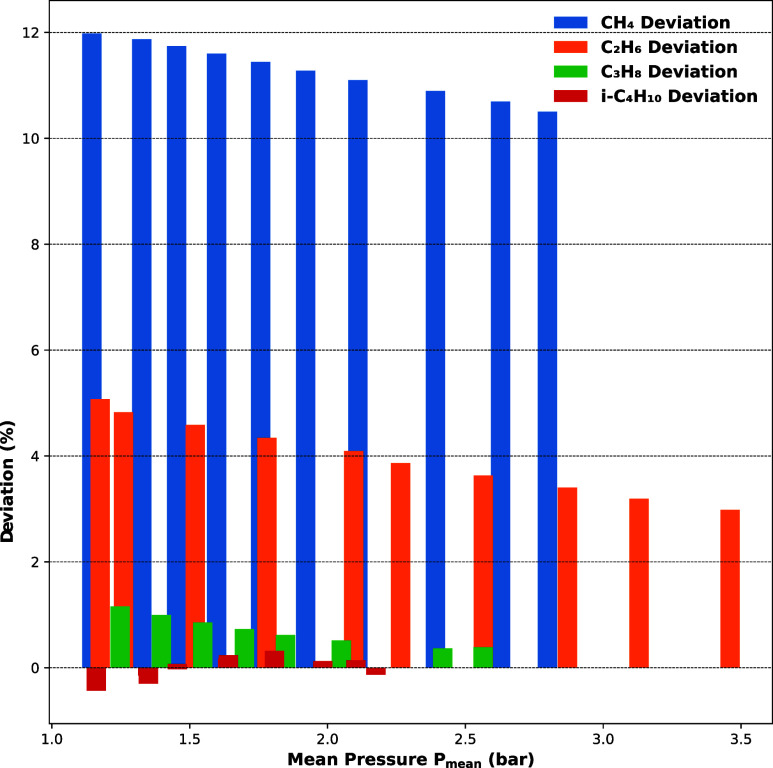
Mass flux deviation between single and double-layer adsorption for different hydrocarbons through anodic alumina (AAO-45 nm).

By equipping the LBM model with proper EOS and analyzing experimental data, we can reproduce the relationship between the strength and range of adhesive strength, density distribution, and mass flow in these confined systems, which is critical for characterizing flow behavior in nanochannels.

## Conclusions

Using a lattice-Boltzmann method to compute the flow of low molecular weight hydrocarbons through straight slits with a width of 45 nm needs a number of adjustments and precautions. The representation of the equation of state within the lattice Boltzmann formulation should be checked against dependence on spatial resolution. Here, it was observed that the densities of saturated liquid or vapor were dependent on lattice spacing, and an adjustable parameter in the equation of state has to be carefully chosen to accurately represent the equation of state in the lattice Boltzmann formulation.

The pressure-driven mass transport of adsorbable species through porous media is generally interpreted as being caused by viscous, molecular and surface flow. Simulations with the lattice-Boltzmann method also show an adsorbed layer at the walls. However, with the usual single-layer interaction term and no-slip boundary condition at the wall, the adsorbed layer has little mobility. The net effect is that an adsorbed layer reduces the total mass flow rate through a pore, because the cross sectional area available to gaseous flow is reduced. With a slip boundary condition, the effect of an adsorbed layer is to increase the total mass flow rate. While both single- and double-layer interaction models yield similar near-wall to bulk density ratios, the two-layer model provides extended density layering in confined geometries. This improvement is most significant for lighter hydrocarbons such as methane, where the double-layer interaction increases mass flux by up to 12% relative to a single-layer formulation. However, for the heaviest hydrocarbon investigated here, isobutane, a further increase of the mass flow rate by using a double-layer interaction term does not occur.

Correct implementation of an equation of state accounting for confinement, consideration of molecular flow in the formulation of interactive forces and the collision operation in the LBM formulation, consideration of adhesive interaction forces that extend over two layers and a slip boundary condition at the wall enables accurate reproduction of experimental permeation data through anodic alumina membranes.
